# Olanzapine-induced Cataract in a Teenage Girl

**DOI:** 10.7759/cureus.2553

**Published:** 2018-04-30

**Authors:** Chang Zhen Lim, Khairy Shamel Sonny Teo, Evelyn Tai

**Affiliations:** 1 Ophthalmology, School of Medical Sciences, Universiti Sains Malaysia, Kubang Kerian, MYS

**Keywords:** olanzapine, schizophrenia, cataract, glucose intolerance, antipsychotic agents, diabetes mellitus

## Abstract

Cataract, defined as cloudiness of the lens, is a common adverse effect of first-generation antipsychotic medications. Newer generation antipsychotics, also known as atypical antipsychotics, are less commonly associated with cataract. A 19-year-old girl with underlying schizophrenia on olanzapine for the past two years complained of gradual blurring of vision in both eyes for four months prior to presentation. On examination, the best corrected visual acuity was counting finger in both eyes. The anterior segment examination showed bilateral diffuse cortical cataract precluding fundus examination. Systemic examination was unremarkable. Blood investigations revealed a high random blood sugar, which normalised after she was initiated on oral hypoglycemic medication. After bilateral lens aspiration, her visual acuity was 6/6 bilaterally. Olanzapine may be cataractogenic via its action as a serotonin antagonist, which results in reduced glucose responsiveness of the pancreatic beta-cells. Patients on anti-psychotic medication are at risk of developing diabetes mellitus and cataract compared to the general population. Screening for diabetes mellitus should be part of the follow-up of these patients. Ophthalmological evaluation is warranted in the presence of visual complaints.

## Introduction

Cataract is the main cause of reversible blindness and serious visual impairment worldwide. It is also notable as a side effect of typical antipsychotic drugs, mainly phenothiazines [1]. Although there is no direct etiological link between atypical antipsychotics and cataract formation, rare cases of cataract have been reported among patients on these medications [1]. We present a case of olanzapine-induced cataract in a teenage girl and discuss the underlying pathophysiology of this condition.

## Case presentation

A physically healthy 19-year-old schizophrenic girl on oral olanzapine 10 mg nightly for the past two years complained of bilateral progressive blurring of vision for four months. She denied any history of eye trauma, red eye, or eye pain. Besides that, she also had polydipsia and nocturia.

**Figure 1:**
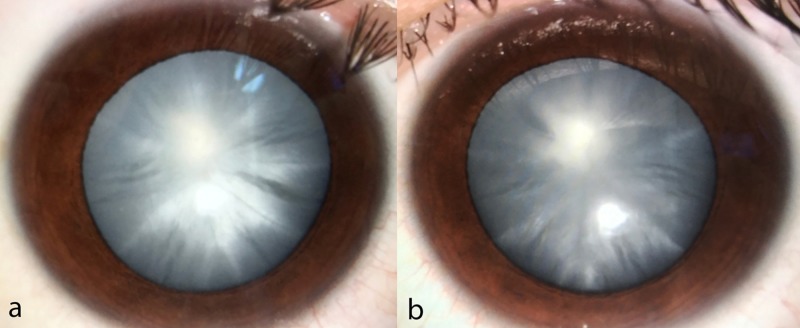
Anterior segment of both eyes Bilateral diffuse cortical cataract in (a) the right eye and (b) the left eye

On examination, visual acuity was counting fingers at 1 m distance in both eyes. Anterior segment examination revealed bilateral diffuse cortical cataract (Figure 1) precluding fundus examination. Ultrasound B-scans of both eyes showed normal posterior segments.

Systemic workup revealed random blood sugar of 21 mmol/L and an HbA1C of 17.1 %. She was referred to an endocrinologist for initiation of treatment to stabilise her blood sugar. The psychiatrist in charge was alerted as well. Both disciplines agreed to substitute oral olanzapine for intra-muscular flupentixol. The patient was also started on oral anti-diabetic medication.

She underwent uneventful bilateral lens aspiration with intraocular lens implantation under general anaesthesia. After the surgery, her best corrected visual acuity was 6/6 in both eyes. She responded well to the new antipsychotic treatment and her blood sugar was well controlled on two types of oral anti-diabetic agents. On her last review, her blood sugar levels had normalised. The fundi were normal.

## Discussion

Atypical antipsychotics are an integral part of schizophrenia management, both for clinical disease control and relapse prevention [2]. Although their side effect profile is more tolerable than their predecessors, potential ocular-associated complications include acute dystonia of extraocular muscles, ocular pigmentation, and cataract [1, 3]. The etiology of antipsychotic-induced cataract is poorly understood, but altered glucose homeostasis secondary to drug-mediated receptor interactions may play a role [4].

Olanzapine is an atypical antipsychotic which mediates its effects by serotonin-dopamine antagonism. It has been hypothesized that serotonin antagonism results in reduced glucose responsiveness of the pancreatic beta-cells [5-6]. This explains olanzepine’s diabetogenic effect, as was observed in our patient. In diabetic patients, impaired glucose tolerance causes sorbitol accumulation in the lens via the polyol pathway, resulting in oxidative stress and cataract formation [7]. Besides that, non-enzymatic glycation of lens proteins may also contribute to formations of cataract [8]. Diabetic cataracts are characterised by diffuse subcapsular or cortical ‘snowflake’ opacities at the initial stage, later followed by generalized cortical cataract, as in our patient [9]. Cortical cataract has been found to be associated with diabetes mellitus regardless of glucose control [10].

Hyperglycemia has been observed as early as 10 days after initiation of olanzapine [6]. Although our patient had symptoms suggestive of a hyperglycemic state for the past few years, she was only diagnosed with diabetes during her pre-operative review. The cataract in our patient may thus be attributed to diabetes mellitus secondary to olanzapine.

## Conclusions

Atypical antipsychotics are the mainstay of schizophrenia management. Cataract is an uncommon side effect of these medications and can occur due to impaired glucose tolerance. Ocular complaints among patients on antipsychotics warrants early ophthalmological and medical evaluation to allow timely treatment of potentially reversible conditions.
